# Mucosal Healing and Fibrosis after Acute or Chronic Inflammation in Wild Type FVB-N Mice and C57BL6 Procollagen α1(I)-Promoter-GFP Reporter Mice

**DOI:** 10.1371/journal.pone.0042568

**Published:** 2012-08-03

**Authors:** Shengli Ding, Kristen L. W. Walton, Randall Eric Blue, Kirk MacNaughton, Scott T. Magness, Pauline Kay Lund

**Affiliations:** 1 Department of Cell and Molecular Physiology, the University of North Carolina at Chapel Hill, Chapel Hill, North Carolina, United States of America; 2 Department of Biology, Missouri Western State University, St. Joseph, Missouri, United States of America; 3 Histology Core Facility, Department of Cell and Molecular Physiology, the University of North Carolina at Chapel Hill, Chapel Hill, North Carolina, United States of America; 4 Division of Gastroenterology and Hepatology, Department of Medicine, University of North Carolina at Chapel Hill, Chapel Hill, North Carolina, United States of America; French National Centre for Scientific Research, France

## Abstract

**Background:**

Injury and intestinal inflammation trigger wound healing responses that can restore mucosal architecture but if chronic, can promote intestinal fibrosis. Intestinal fibrosis is a major complication of Crohn’s disease. The cellular and molecular basis of mucosal healing and intestinal fibrosis are not well defined and better understanding requires well characterized mouse models.

**Methods:**

FVB-N wild type mice and C57BL6 procollagen α1(I)-GFP reporter mice were given one (DSS1) or two (DSS2) cycles of 3% DSS (5 days/cycle) followed by 7 days recovery. Histological scoring of inflammation and fibrosis were performed at DSS1, DSS1+3, DSS1+7, DSS2, DSS2+3, and DSS2+7. Procollagen α1(I)-GFP activation was assessed in DSS and also TNBS models by whole colon GFP imaging and fluorescence microscopy. Colocalization of GFP with α-smooth muscle actin (α-SMA) or vimentin was examined. GFP mRNA levels were tested for correlation with endogenous collagen α1(I) mRNA.

**Results:**

Males were more susceptible to DSS-induced disease and mortality than females. In FVB-N mice one DSS cycle induced transient mucosal inflammation and fibrosis that resolved by 7 days of recovery. Two DSS cycles induced transmural inflammation and fibrosis in a subset of FVB-N mice but overall, did not yield more consistent, severe or sustained fibrosis. In C57BL6 mice, procollagen α1(I)-GFP reporter was activated at the end of DSS1 and through DSS+7 with more dramatic and transmural activation at DSS2 through DSS2+7, and in TNBS treated mice. In DSS and TNBS models GFP reporter expression localized to vimentin^+^ cells and much fewer α-SMA^+^ cells. GFP mRNA strongly correlated with collagen α1(I) mRNA.

**Conclusions:**

One DSS cycle in FVB-N mice provides a model to study mucosal injury and subsequent mucosal healing. The procollagen α1(I)-GFP transgenic provides a useful model to study activation of a gene encoding a major extracellular matrix protein during acute or chronic experimental intestinal inflammation and fibrosis.

## Introduction

Injury of the intestinal epithelium and acute or chronic inflammation trigger wound healing responses involving activation of mesenchymal cells and increased extracellular matrix (ECM) synthesis, remodeling or deposition [1]–[3]. Ideally, wound-healing responses after acute injury or inflammation will lead to mucosal healing and restoration of normal tissue architecture. However, chronic or recurring intestinal injury and inflammation can promote excessive transmural wound-healing responses that lead to fibrosis. Fibrosis is characterized by excessive ECM deposition, with procollagen α1(I) representing a prominent ECM component, and is often accompanied by distortion of normal tissue architecture and dysfunction.

Intestinal fibrosis can occur in response to chronic inflammatory bowel diseases (IBD) including ulcerative colitis (UC) or Crohn’s Disease (CD) but is most common and problematic in CD [1], [3]. In CD, chronic inflammation is typically transmural, extending through the mucosal layer to underlying submucosa, muscularis propria and serosa where mesenchymal cells, which are the major sources of ECM, reside [1], [4]. Fibrostenotic disease, typified by excessive ECM deposition, thickening of the bowel wall, and narrowing or stenosis of the intestinal lumen affects approximately one third of patients with CD [5]. Despite major progress in development of anti-inflammatory therapies in CD, progress on development and clinical testing of anti-fibrotic agents has been slower. The major treatment for fibrosis in CD is surgical intervention yet disease and fibrosis recur in many CD patients necessitating additional surgeries [3], [5]. The lack of effective medical therapies for intestinal fibrosis in part reflects a limited understanding of the pathophysiology and molecular and cellular mediators of wound healing and fibrosis in the intestine. Animal models have made major contributions to our understanding of the immunological and genetic mechanisms associated with IBD and have contributed to the development and testing of anti-inflammatory therapies. The goal of the current study was to characterize animal models that may be useful for comparisons of mesenchymal responses associated with mucosal healing after acute injury and inflammation with mesenchymal responses to chronic transmural injury that lead to fibrosis. Such models are desirable as systems to aid better understanding of mechanisms that underlie normal mucosal healing versus fibrosis and to test interventions that may prevent or limit fibrosis.

DSS-induced injury and inflammation of the colon has several attractive features including the technical ease of the model since DSS is given in drinking water, the lack of requirement for anesthesia during treatment and existing information that DSS induces acute mucosal injury and inflammation in mice on multiple genetic backgrounds [6]–[9]. We previously reported that mice on a mixed C57BL6/SJL background show mucosal repair and disease recovery during 10 days after a single cycle of DSS [10]. Okayasu et al. reported that oral administration of multiple cycles of 5% DSS in drinking water of BALB/c mice was able to induce chronic intestinal inflammation [9]. The current study examined the impact of one or two cycles of DSS followed by recovery periods in two commonly used strains of mice, FVB-N and C57BL6 mice. Our aim was to examine if either or both strains exhibit mesenchymal cell activation associated with mucosal healing after one cycle of DSS treatment, or develop transmural or more severe or sustained fibrosis after a second DSS cycle or a subsequent recovery period. FVB-N mice were tested because transgenic or gene deletion models are commonly derived on this genetic background. Our findings suggest that this genetic strain of mice exhibits increases in ECM deposition early during recovery from a single DSS cycle followed by mucosal repair responses that normalize mucosal architecture. In FVB-N mice, two DSS cycles did not result in more consistent, sustained or severe transmural fibrosis although transmural disease was observed in a subset of animals. Thus DSS colitis in this genetic background provides opportunities to study mesenchymal responses during acute mucosal injury and mucosal healing.

Fibrogenic responses to DSS have been previously reported in mice on the C57BL6 background [11], [12]. Our studies examined responses to one or two DSS cycles in C57BL6 mice expressing a procollagen α1(I) promoter-GFP reporter transgene to test if this model may be useful to assess intestinal mesenchymal responses to acute or chronic intestinal inflammation. We demonstrate that the reporter is activated during acute or chronic DSS-induced inflammation and provides a useful tool to visualize, quantify and define cell types that exhibit activation of gene encoding, a major component of ECM during fibrosis. Activation of the procollagen α1(I)-GFP reporter was also confirmed in the TNBS model of chronic T-cell mediated colitis. In addition, our studies revealed that compared with females, males on both the FVB-N and C57BL6 backgrounds showed more limited ability to resolve disease and had increased mortality after one or two DSS cycles. This information is important for appropriate design and interpretation of future studies of DSS colitis and associated wound healing or fibrogenic responses that may include male and female mice.

## Materials and Methods

### Ethics Statement

All studies were approved by the Institutional Animal Care and Use Committee (IACUC) of the University of North Carolina at Chapel Hill (Approved protocol number: 10–197).

### Animal Models

Wild Type (WT) FVB-N mice were obtained from a colony maintained in our specific pathogen-free (SPF) facility. Procollagen α1(I) promoter-green fluorescent protein (GFP) transgenic mice (procollagen α1(I)-GFP reporter mice) on a mixed C3H/C57BL6 background were provided by Dr. David Brenner (University of California, San Diego) and were back-crossed with C57BL6 mice for more than 10 generations to generate mice on the inbred C57BL6 background. These mice contain the basal promoter of the murine procollagen α1(I) gene (−3122 to +111 relative to transcription start site +1) and two upstream DNase sensitive sites that are important for maximal expression [13].

α-SMA (smooth muscle actin promoter)- *Discomsoma sp.* red fluorescence protein (RFP) transgenic mice on CD1 background were provided by Dr. Scott Magness and were generated as previously described [14]. α-SMA-RFP mice were back-crossed with C57BL6 mice for more than 5 generations to generate mice on C57BL6 background. Both procollagen α1(I)-GFP reporter mice and α-SMA-RFP mice were maintained as heterozygotes and were cross-bred to generate procollagen α1(I)-GFP/α-SMA-RFP dual reporter mice. All genotyping of procollagen α1(I)-GFP, α-SMA-RFP or α-SMA-RFP/procollagen α1(I)-GFP mice was conducted by observing fluorescence in a small tail-snip.

### Dextran Sodium Sulfate (DSS) Treatment

DSS of molecular weight 30,000–40,000 was purchased from TDB Consultancy (Uppsala, Sweden). DSS was dissolved in dH_2_O to a concentration of 3% wt/vol and changed daily to prevent bacterial growth. Adult FVB-N mice (n≥5 per time point) and C57BL6 procollagen α1(I)-GFP reporter mice (n = 2 per time point) were given one or two cycles of 3% DSS (one cycle = 5 days of 3% DSS) with seven days of recovery between cycles. Animals were studied at the end of one or two cycles of DSS treatment (DSS1 or DSS2), and at 3 or 7 days after one (DSS1+3, DSS1+7) or two (DSS2+3, DSS2+7) cycles of DSS. These time points were chosen to assess if acute mucosal injury by one cycle of DSS led to mucosal healing during 7 days of recovery from a single DSS cycle and if two cycles of DSS led to increased severity of fibrosis or transmural fibrosis. Age and sex-matched control animals on each background were given water instead of DSS and were studied in parallel with treated animals. Animals were weighed daily and fecal occult blood test was performed every two days to provide overt measures of disease. Animals were euthanized if they lost more than 15% body weight or if they became lethargic, immobile or failed to eat or drink. Data on survival reflects percentage of animals that did not require euthanasia prior to the study end point. Histological analyses were performed only on animals that survived to the assigned study endpoint. Follow-up pilot studies were performed on dual procollagen α1(I)-GFP/α-SMA-RFP reporter mice (n = 2) given 1 or 2 DSS cycles to test whether the α-SMA-RFP reporter might provide advantages over antibody based methods in defining whether cells exhibiting procollagen α1(I)-GFP also express α-SMA.

### Sample Collection

Animals were killed by intraperitoneal injection of an overdose (150 mg/kg body weight) of sodium pentobarbital (Nembutal, Ovation Pharmaceuticals Inc. Deerfield, US) and the entire colon was dissected and flushed with 1X phosphate buffered saline (PBS)(pH = 7.4). In FVB-N mice, the distal half of the colon was dissected for analyses since preliminary studies indicated that this region of colon is most affected. Since it is known that DSS-induced injury can be focal, the distal half of the colon was split into three segments for histology as follows: D1(proximal 1/3 of distal colon), D2 (mid 1/3 of distal colon) and D3(distal 1/3 of distal colon). Segments D1 and D2 were fixed in 4% paraformaldehyde (PFA) and Bouin’s fixative respectively and paraffin embedded. Segment D3 was oriented in O.C.T. (Tissue-Tek) and frozen on dry ice and isopentane. Different fixation conditions were used to provide samples compatible for future immunostaining studies that may require different fixatives. Sections from D1 and D3 were routinely used for histological scoring of inflammation and fibrosis on these two independent segments. D2 was used only if histology was suboptimal in D1 or D3 of a given animal. For procollagen α1(I)-GFP reporter mice and procollagen α1(I)-GFP/α-SMA-RFP mice, whole colon was dissected and imaged for GFP then fixed in 4% PFA, cryoprotected in sucrose and embedded in O.C.T. for histology or immunofluorescence. A small segment at the distal end of the proximal half of the colon was frozen for RNA extraction.

### Histological Assessment of Inflammation and Fibrosis in FVB-N Mice

The distal colon was evaluated as this is the most severely affected colonic segment in DSS-induced colitis [6], [9]. Severity of colitis was scored on H&E stained colon sections by a blinded observer [10]. Sections from segments D1 and D3 were evaluated for each animal and an average of scores in the two segments was used as the score for each individual animal. Segment D2 was scored only on a subset of animals if tissue quality of other segments was poor. Briefly, inflammation severity, inflammation extent and crypt loss were each given a score ranging from 0–4 and each score was multiplied by the extent of the section involved (0 = 0; ≤25% = 1, ≤50% = 2; ≤75% = 3 and ≤100% = 4). Total colitis score is the sum of the scores for inflammation severity, inflammation extent and crypt loss and was calculated as the average of total colitis scores from D1 and D3 in a given animal. Fibrosis was scored by a blinded observer on adjacent sections from segments D1 and D3 stained with Masson’s Trichrome (stains collagen and proteoglycans) or Sirius red (stains fibrillar collagen). The scoring criteria used for fibrosis are shown in Table 1 and fibrosis score for each animal is the average of scores for segments D1 and D3. Histological evaluation and scoring was performed using a Nikon Microphot-FXA microscope equipped with an Optronics DEI 750-3-chip CCD camera for digital imaging.

**Table 1:** Histological fibrosis scoring method.

Category	Score	Description
Collagen (fibrosis)	0	No increase
	1	Increased in the submucosa
	2	Increased in the mucosa
	3	Increased in the muscularis mucosa; thickening/disorganization of the muscularis mucosa
	4	Increased in the muscularis propria (evident increases in collagen fibrils for Sirius red)
	5	Gross disorganization of the muscularis propria; increased Sirius red/GFP or thickening of the serosa
Percent involvement	1	1–25% of section
	2	26–50% of section
	3	51–75% of section
	4	76–100% of section

### Enhanced GFP (EGFP) Imaging in DSS Treated Procollagen (α1)I-GFP Mice

Whole colon was dissected and immediately imaged using a charge-coupled device camera in a light-tight imaging box with a dual-filtered light source and emission filters specific for EGFP (LT-99D2 Illumatools; Lightools Research, Encinitas, CA). Identical exposure times were used to capture images from each animal.

### Real Time PCR Analyses

Total RNA was extracted from segment of colon from each DSS treated procollagen α1(I)-reporter animal using the RNeasy Mini Kit (Qiagen) according to manufacturer’s instructions. Reverse-transcription was performed using AMV-RT (Promega). Quantitative real time PCR analyses were completed on the Rotorgene 2000 (Qiagen) using Invitrogen Platinum qPCR Supermix-UDG and the following Taqman primer-probe sets (Applied Biosystems): mouse collagen α1(I) Mm00801666_g1, customized mouse EGFP Taqman primers (Forward: 5′-AGTCCGCCCTGAGCAAAGA-3′, Reverse: 5′-TCCAGCAGGACCATGTGATC-3′) and probe (5′ 6-FAM-CCCAACGAGAAGCG-MGB-3′). 18 S Mm03928990_g1was used as an invariant control. Non-reverse transcribed samples were used as negative controls. Collagen α1(I) mRNA in each sample was normalized to 18 S mRNA.

### Immunofluorescence Staining

To establish mesenchymal cell types that exhibited procollagen α1(I)-reporter expression, cellular sites of GFP expression were compared with sites of vimentin or α-smooth muscle actin (α-SMA) using immunofluorescence. Before the primary antibody incubation, frozen sections were pretreated with 0.05 M Tris Triton (TT) epitope retrieval solution at room temperature (RT) for 30 minutes and blocked in 5% normal goat serum (NGS) diluted in TT buffer for 1 hr and subsequently incubated with primary antibodies (α-SMA, Cat#04–1094, 1∶200, Millipore, Billerica, MA; Vimentin, Cat# NBP-140730, 1∶500, Novus Biologicals, Littleton, CO) that were diluted in 5% NGS/TT buffer overnight at 4°C. Detection of primary antibodies was performed with secondary antibodies (Goat anti mouse Cy3, Cat#115–165–166, 1∶500; Goat anti Rabbit Cy3, Cat#111–165–144, 1∶500, Jackson Immuno) for 1 hr at RT. Nucleic acid staining was carried out by labeling with bisbenzamide for 10 minutes. Following three washes with 0.05 M Tris buffer, coverslips were mounted in gel-mount (Electron Microscopy Sciences, Hatfield, PA). Photomicrographs were collected using a Nikon Eclipse E800 (Melville, NY) fluorescence microscope. Procollagen α1(I)-GFP and α-SMA-RFP activation were detected by fluorescence microscopy.

### TNBS Induced Colitis

Procollagen α1(I)-GFP reporter mice were treated with TNBS ethanol to induce colitis according to the method reported by Wirtz [15]. TNBS treatment is known to activate T cells even during periods of early acute inflammation [15], [16]. We therefore used this model to test if the procollagen α1(I)-GFP reporter is activated in response to T cell mediated colitis. Briefly, on day 1, mice were presensitized with 150 µl TNBS presensitization solution (4 volumes of acetone/olive oil with 1 volume of 5% TNBS to obtain 1% (w/v) TNBS) administered on shaved skin on the back of the animal. Control animals were given presensitization solution without TNBS. Eight days later, the mice were anesthetized under isofluorane and given TNBS/ethanol (2.5 mg/mouse TNBS +50% ethanol, 0.1 ml) by enema. Control animals were given enema with only 50% ethanol or water. Mice were euthanized five days later. Whole colon was dissected, GFP was visualized and then colon was fixed in 4% PFA, cryoprotected in sucrose and embedded in O.C.T. for histological analyses.

### Statistical Analysis

Values are expressed as mean ± standard error (SE) or presented as values for individual animals. Mean histological scores were analyzed by one-way ANOVA followed by pair wise comparisons using Tukey’s post-hoc test. Statistical analyses of histological inflammation and fibrosis include only females since few males survived to study endpoint. Regression analyses were performed to test for correlations between histological measures of inflammation and fibrosis or procollagen α1(I)-GFP activation. Regression analysis was also performed to test for correlations between EGFP mRNA and collagen α1(I) mRNA in colon of C57BL6 procollagen α1(I)-GFP reporter mice. A *p* value <0.05 was considered statistically significant.

## Results

### Clinical Indices of Disease in FVB-N Mice and Collagen-GFP B6 Mice

As a group, FVB-N mice tolerated 1 or 2 cycles of 3% DSS followed by recovery with an overall survival rate of 86%. However, over the course of treatment, survival of female mice was greater (98%) than survival of males (73%). Of the male mice that did not survive until the intended study time, 73% required euthanasia during the first recovery period, and the remaining 27% required euthanasia during the second cycle of DSS or subsequent recovery period. None of the male mice originally designated for study at DSS2+7 survived until that study time. Therefore, data obtained at this time point reflects only female mice.

**Figure 1:**
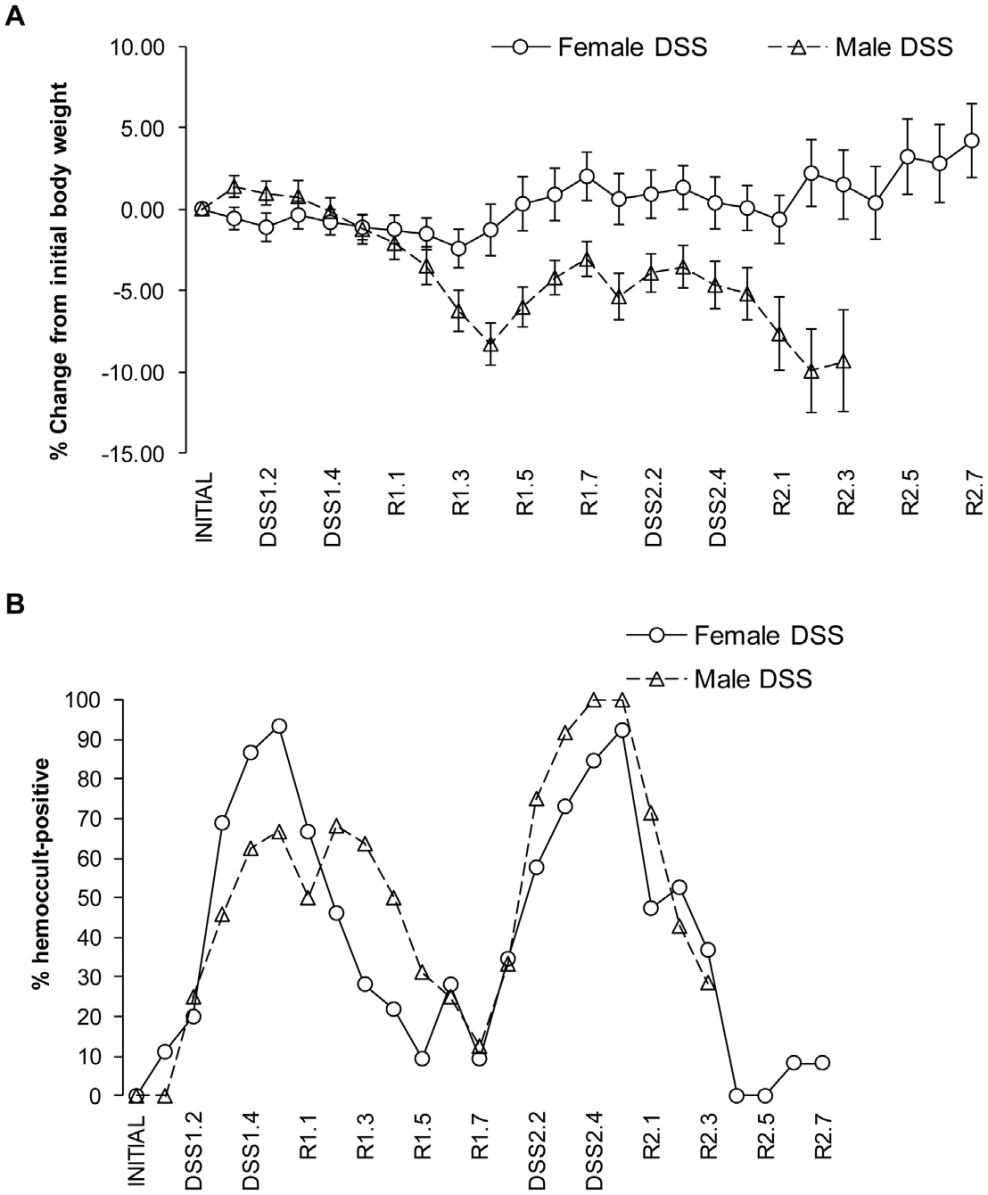
DSS treated male FVB-N develop more severe disease that females. (A) Percentage body weight change in male and female FVB-N mice after one or two cycles of DSS treatment. (B) Percentage of hemoccult positive male and female FVB-N mice treated with DSS. Data are presented as mean ± SEM of 6–14 mice per treatment group. DSS1: one cycle of DSS; DSS2: two cycles of DSS; R1: recovery period after one cycle of DSS; R2: recovery period after two cycles of DSS.

Consistent with the higher survival observed in female FVB-N mice, DSS treatments were associated with minimal weight loss in female FVB-N mice and more dramatic weight loss in male FVB-N mice. As shown in Figure 1A, males given DSS showed a decline in body weight during the first 4 days after one cycle of DSS treatment. Those male mice that survived then began to regain weight but male mice given a second cycle of DSS showed a progressive decline in body weight throughout the second DSS cycle and recovery. Hemoccult testing was used as a measure of bleeding associated with active injury and inflammation induced by DSS. Virtually 100% of females became hemoccult positive by the end of DSS treatment, but then this rapidly declined back to baseline after 7 days of recovery indicative of mucosal healing (Figure 1B). A similar pattern was observed after 2 DSS cycles (Figure 1B). Consistent with greater disease susceptibility in males, a larger proportion of the males that did not require euthanasia remained hemoccult positive during the initial 7 days of recovery after one DSS cycle (Figure 1B).

### FVB-N Mice Show Mucosal Healing after One DSS Cycle but not Sustained Fibrogenic Responses after Two DSS Cycles

**Figure 2:**
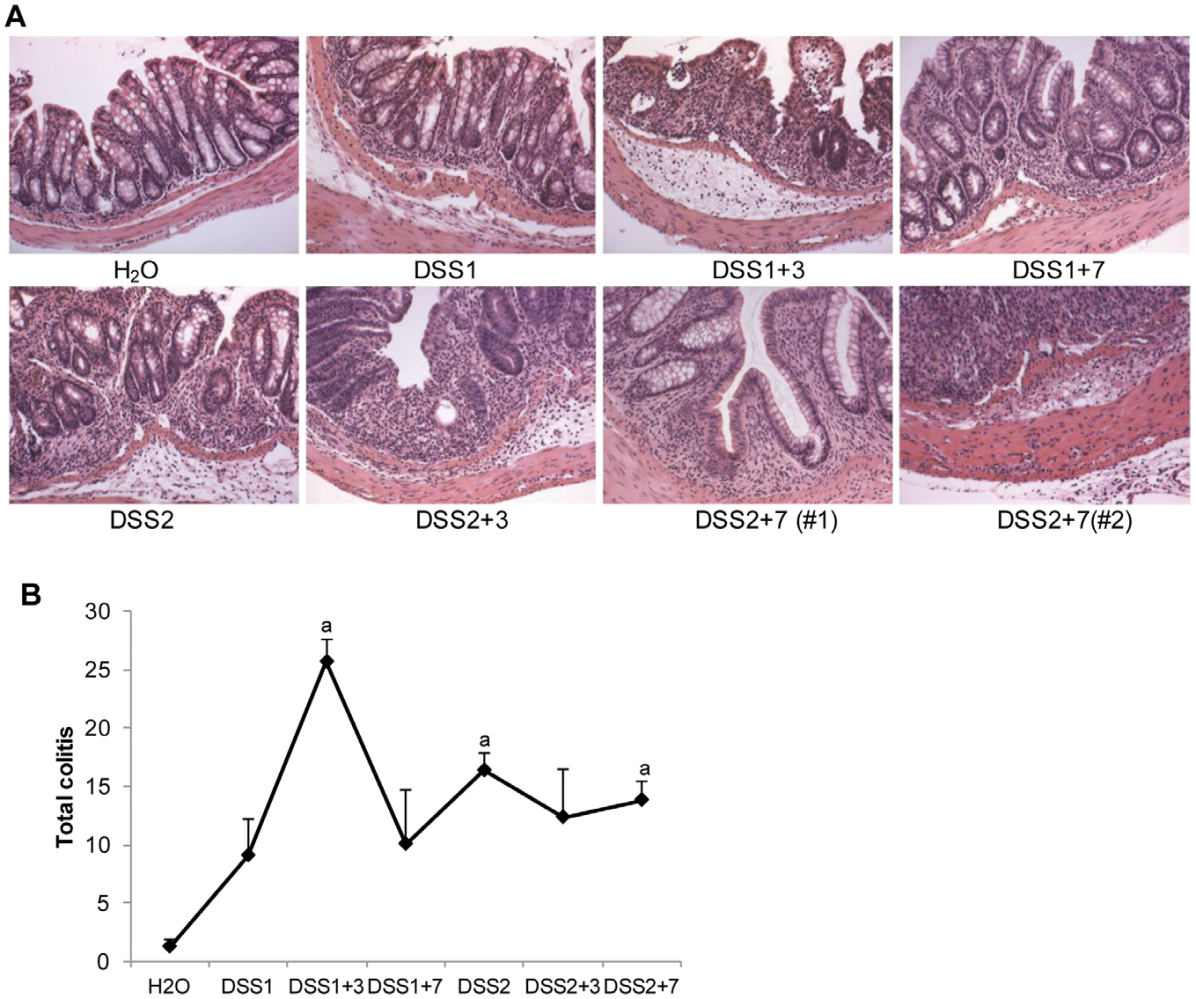
Two cycles of DSS treatment induce sustained, severe and transmural inflammation in FVB-N mice colon. (A) Representative photomicrographs of H&E staining on distal colon of FVB-N mice after DSS treatment (10X). (B)Total colitis score in colon of DSS-treated FVB-N mice. Data are presented as mean ± SEM. a: *p*<0.05 versus scores in water control mice.

Figure 2A shows representative H&E stained sections at different times after one or two DSS cycles and illustrates that inflammation was largely confined to the mucosa at the end of one DSS cycle, became most severe and extended into submucosa at DSS1+3 and then partially resolved after 7 days of recovery. Total colitis scores (Figure 2B) provide quantitative evidence that inflammation and mucosal damage were maximum at DSS1+3. By DSS1+7, mean colitis scores did not differ significantly from water controls, indicating that mucosal healing and resolution of inflammation occurred in a majority of animals by this time point. After two DSS cycles, inflammation extended to the submucosa by DSS2+3 and in a subset of animals, extended through the muscularis propria and serosal layers by DSS2+7 (Figure 2A). Mean colitis scores were significantly elevated above water controls at DSS2+7 but were not as high as observed at DSS1+3.

**Figure 3:**
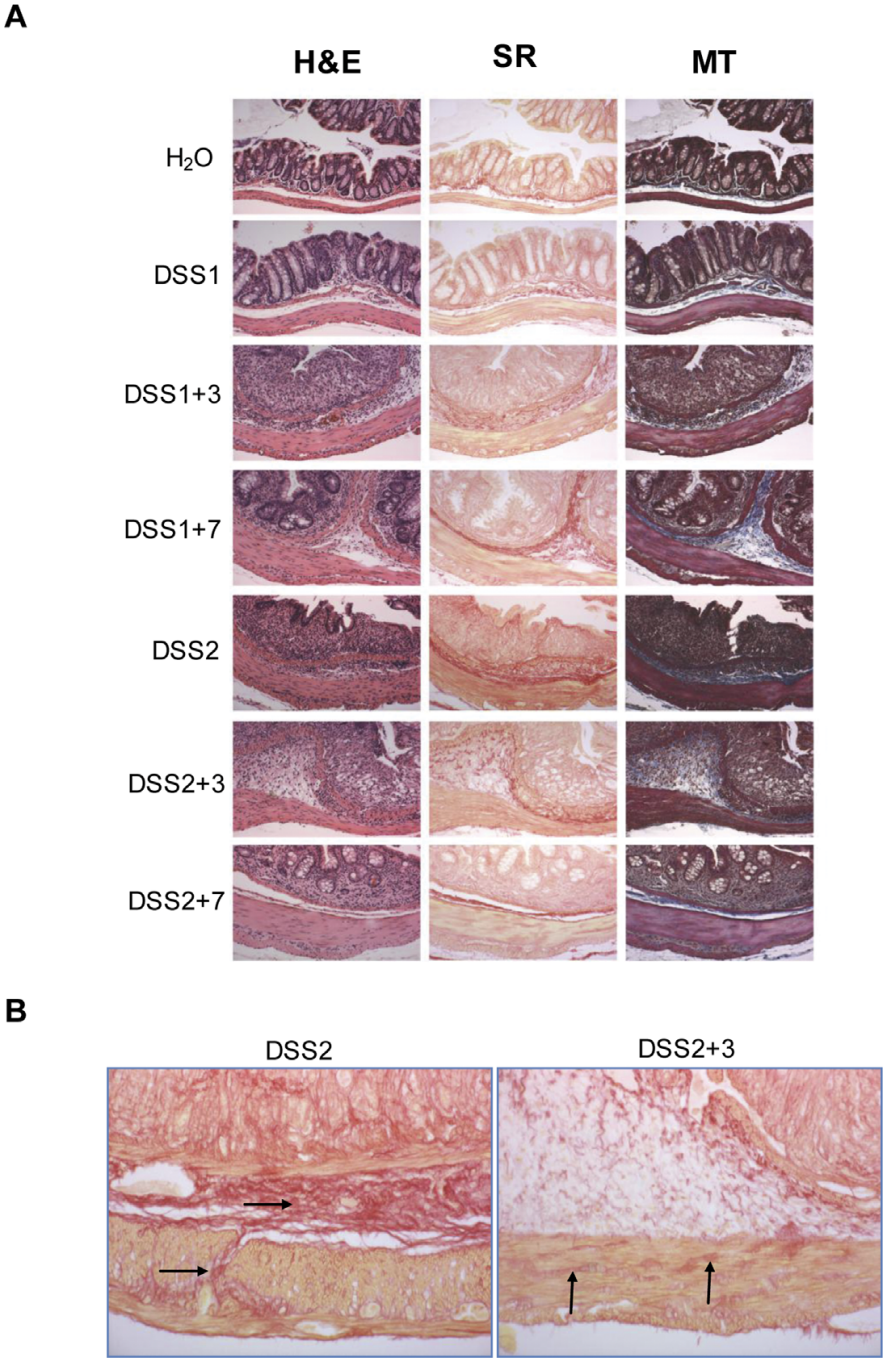
Histological analysis demonstrate induction of submucosal and transmural collagen deposition in DSS treated mice colon. (A) Hemotoxylin and eosin (H&E), Sirius red staining (SR) and Masson’s Trichrome staining (MR) on FVB-N mice colon at different time points during DSS treatments (10X). (B) High power photomicrographing of Sirius red staining in sections of colon from FVB-N given 2 DSS cycles to illustrate muscularis and transmural collagen deposition (black arrows) (20X).

Representative Masson’s Trichrome- and Sirius red-stained sections are shown in Figure 3, along with adjacent sections stained with H&E. Increased ECM deposition was apparent in the lamina propria, especially in regions of severe crypt damage and inflammation. The most dramatic increases in ECM deposition occurred in the submucosa, with strong collagenous bands evident adjacent to the muscularis mucosa during recovery from one cycle of DSS treatment. After 2 DSS cycles, a subset of animals showed transmural fibrosis with increased ECM being evident in submucosa, muscularis propria and serosa (Figure 3B).

**Figure 4:**
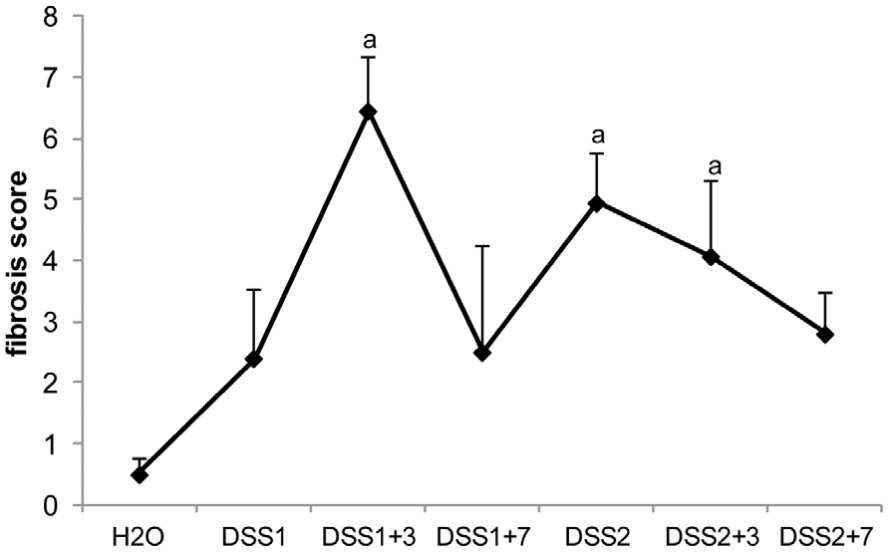
Two cycles of DSS did not induce more severe fibrogenic responses in FVB-N mice. Fibrosis scores in colons of FVB-N mice at different time points during DSS treatment. Data are presented as mean ± SEM of 6–10 mice per treatment group. a: *p*<0.05 versus scores in water control mice.

Fibrosis was scored by a blinded observer using the semi-quantitative scheme outlined in Table 1. Mean fibrosis scores in each group are shown in Figure 4. Post-hoc analyses revealed that the mean fibrosis score was significantly elevated above water controls at DSS1+3, DSS2 and DSS2+3. However, the mean fibrosis scores at DSS1+7 and DSS2+7 were not significantly greater than water controls. This suggests that increased ECM after one or two DSS cycles is transient and followed by a return towards normal levels within 7 days after DSS. Regression analysis on colitis scores and fibrosis scores across all time points indicated a strong and highly significant correlation (r^2^ = 0.82, *p*<0.005).

### Procollagen α1(I)-GFP Mice as a Useful Reporter for Intestinal Fibrosis

We assessed procollagen α1(I)-GFP mice after one or two DSS cycles and after TNBS treatment to test if this model may provide a useful reporter for mesenchymal cell activation and fibrosis. Since these mice are on a C57BL6 background, this allowed us to test whether the gender specific differences in disease severity observed in DSS-treated FVB-N mice were also observed in mice on the widely used C57BL6 genetic background. As shown in Table 2, female C57BL6 mice lost less weight than males over the course of two DSS cycles and recovery. In addition, one third of the males tested required euthanasia while all females survived. Thus, across two separate genetic backgrounds, males are more susceptible to DSS colitis than females.

**Table 2:** Body weight loss and survival rate of DSS treated C57BL6 mice.

Average body weight loss					Survival rate After DSS2
	DSS1	DSS1+7	DSS2	DSS2+7	
Control (M+F, n = 3)	0	0	0	0	100%
Female (n = 4)	2%	5.5%	7.2%	3.5%	100%
Male (n = 3)	2%	21.5%	17.3%	*	66%

* did not measure due to high mortality/need for euthanasia.

(Data reflect non-transgenic littermates as well as procollagen α1(I)-GFP mice at each time).

**Figure 5:**
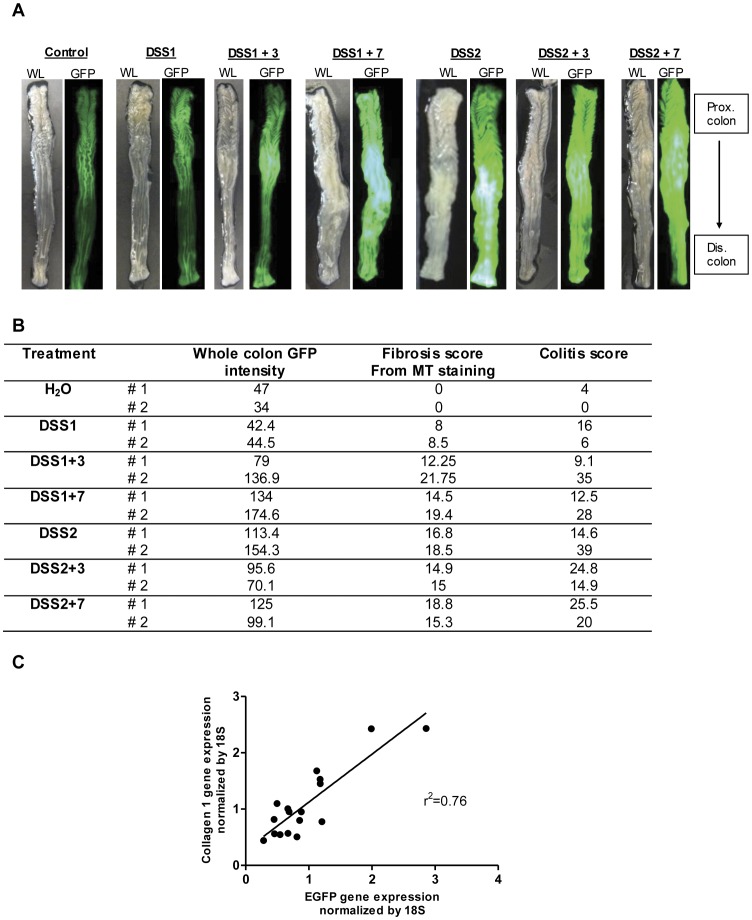
Procollagen α1(I)-GFP reporter expression is induced in mice colon after DSS treatment and recovery. (A) Representative white light and GFP images of whole colon from collagen GFP reporter mice after DSS treatment. (B) Quantitative data for whole colon intensity, fibrosis score from Masson’s Trichrome (MT) staining and colitis score for each animal (n = 2 per treatment group and time point). (C) Regression analysis on EGFP mRNA and collagen α1(I) mRNA in colon of C57BL6 procollagen α1(I)-GFP reporter mice(r^2^ = 0.76, *p*<0.001).

**Figure 6:**
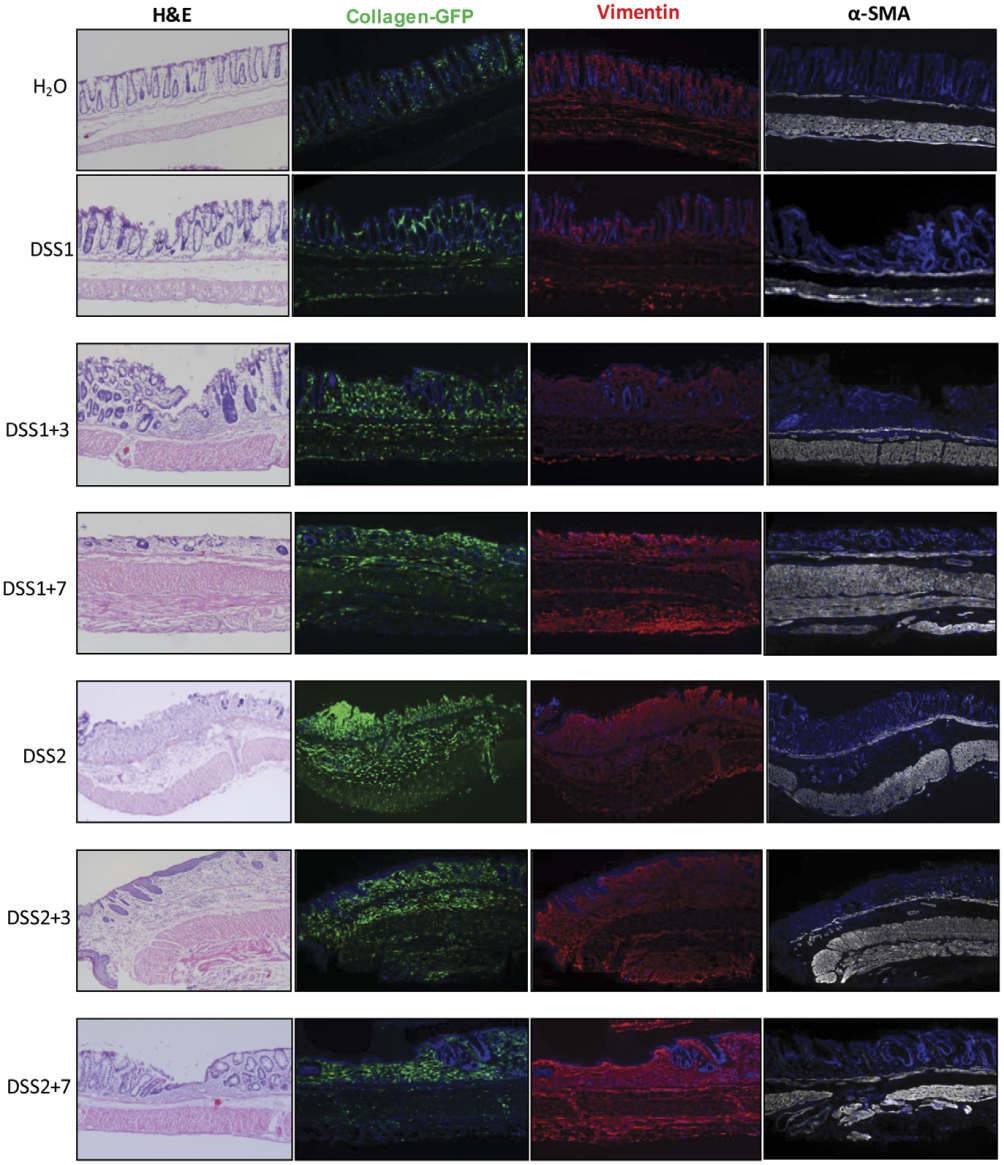
Low power images of H&E, GFP, vimentin and α-SMA staining from DSS treated mice colon (4X). This indicates progressively greater and transmural activation of the procollagen α1(I)-GFP reporter after 2 DSS cycles. Both GFP and vimentin positive cells were expanded within mucosa, submucosa and muscularis propria and serosa. However, α-SMA antibodies stained primarily muscularis mucosa and muscularis propria.

As shown in Figure 5, evaluation of entire colon from water controls or DSS treated mice revealed that collagen GFP expression was obviously and dramatically induced in mid and distal colon by DSS1+7 and at all time points after 2 DSS cycles (Figure 5A). White light pictures of the same colon tissue demonstrate enlarged mid and distal colon at DSS1+7 and subsequent times. H&E staining (Figure 6) demonstrated that in contrast to FVB-N mice, C57BL6 mice do not show mucosal healing during the 7 days following one DSS cycle. Inflammation and crypt loss progressively worsened from the end of one cycle of DSS up to DSS1+7 and a second DSS cycle led to very severe transmural inflammation which did not resolve by DSS2+7. Evaluation of GFP on the same sections as stained with H&E (Figure 6) revealed that the procollagen α1(I)-GFP reporter was activated at the end of one DSS cycle and through 7 days of recovery with induction primarily in lamina propria, submucosa and scattered cells within muscularis propria and serosa. After two DSS cycles, maximal intense and transmural activation of collagen GFP was still apparent through DSS2+3 and DSS2+7. To establish if procollagen α1(I)-GFP activation could provide a semi-quantitative measure of fibrosis, whole colon GFP intensity was assessed using ImageJ 1.43u, NIH, USA. A histologic fibrosis score was obtained from Masson’s Trichrome stained sections of distal colon using the fibrosis scoring criteria listed in Table 1. Colitis score was obtained on H&E sections from the same colon segment (n = 2 for each time point). Whole colon GFP intensity, fibrosis score from Masson’s Trichrome staining and colitis scores were elevated from DSS1+3 onwards (Figure 5B). Regression analysis revealed that whole colon GFP intensity correlated significantly with a histological fibrosis score based on Masson’s Trichrome staining (r^2^ = 0.742, *p*<0.001) and with histological colitis score (r^2^ = 0.669, *p* = 0.002). The histological fibrosis score based on Masson’s Trichrome staining also significantly correlated with colitis score (r^2^ = 0.738, *p*<0.0001). Regression analysis (Figure 5C) showed strong and significant correlation between GFP mRNA expressed due to activation of the procollagen α1(I)-GFP transgene and endogenous collagen α1(I) mRNA (r^2^ = 0.76, *p*<0.001), providing evidence that the procollagen α1(I)-GFP reporter provides a valid measure of injury or inflammation induced activation of a gene encoding a major component of fibrotic ECM. Furthermore, the strong correlation between whole colon GFP and fibrosis scores provides evidence that imaging GFP on whole colon could be useful and convenient to evaluate or test impact of genetic or therapeutic interventions on procollagen α1(I)-GFP expression associated with acute wound healing responses or fibrosis, especially in high throughput studies.

### One or Two DSS Cycles Induce Procollagen α1(I)-GFP Reporter Expression in Cells Expressing Antigenic Markers of Fibroblasts and Myofibroblasts

**Figure 7:**
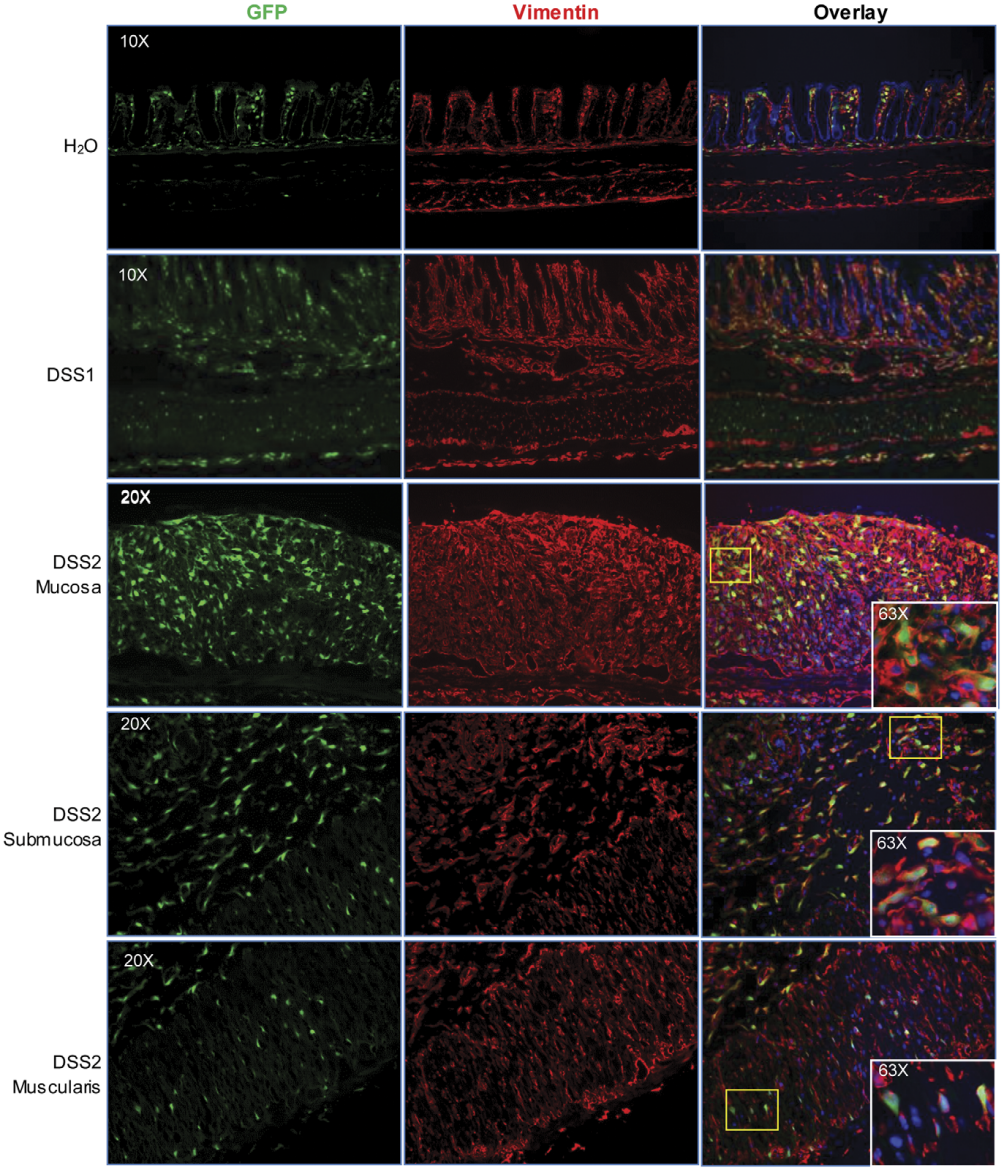
High power images of colocalization vimentin(red) and procollagen α1(I)-GFP (green) on DSS treated mice colon. Overlay (yellow cells, high power) demonstrates extensive colocalization of vimentin and GFP.

**Figure 8:**
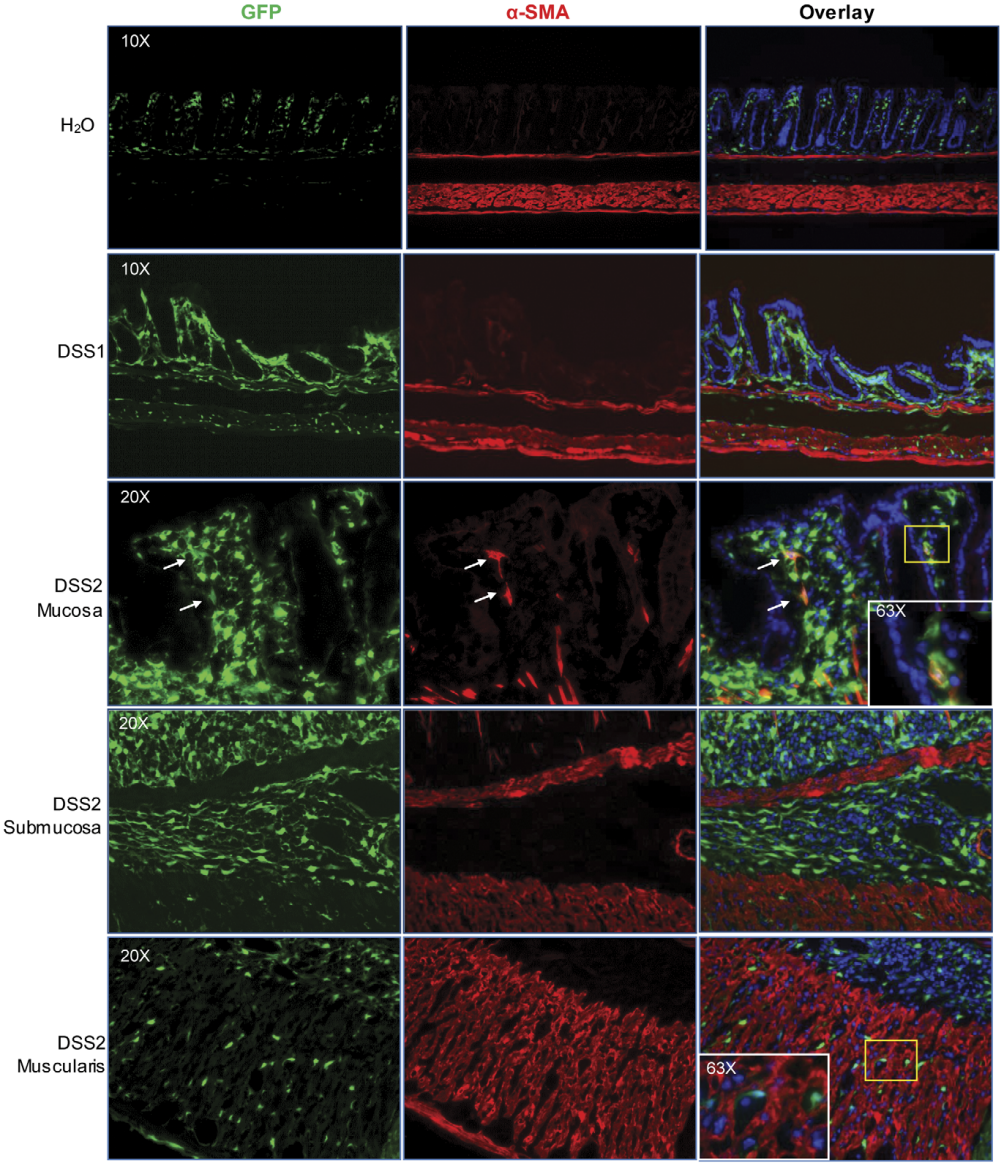
High power images of colocalization α-SMA (red) and procollagen α1(I)-GFP(green) on DSS treated mice colon. Overlay demonstrates very limited co-localization in mucosa (white arrows). No colocalization was detected in submucosa and muscularis.

Multiple fibrogenic mesenchymal cells including fibroblasts (vimentin^+^/α-SMA^−^), myofibroblasts (vimentin^+^/α-SMA^+^) and smooth muscle cells (vimentin^−/^α-SMA^+^) have been implicated in intestinal fibrosis [1]. The localizing of GFP was compared with either vimentin or α-SMA on the adjacent sections, and fluorescence signals were compared with H&E stained adjacent sections to reveal histology. Qualitative evaluation of low power images (Figure 6) demonstrates that in the 7 days following one or two DSS cycles, there is a dramatic expansion of both GFP and vimentin positive cells within mucosa, submucosa and disorganized muscularis propria and serosa. In contrast, α-SMA antibodies stained primarily muscularis mucosa and muscularis propria. Colocalization of GFP with vimentin or α-SMA was further assessed by examination of sections under high power magnification. As shown in Figure 7, the number of cells showing colocalization of GFP and vimentin was dramatically increased after DSS treatment, especially in the mucosa and submucosa. After 1 or 2 cycles of DSS, the majority of mucosal and submucosal GFP^+^ cells are also vimentin^+^ with scattered GFP^+^/vimentin^+^ cells were also evident in muscularis and present in serosa. Figure 8 also shows there are relatively few GFP^+^ cells which colocalize with α-SMA^+^ in water controls or after one DSS cycle. After two DSS cycles, a few double positive GFP^+^/α-SMA^+^ cells (myofibroblasts) were found in mucosa, but the GFP^+^ cells in submucosa or muscularis layers did not show positive α-SMA-immunofluorescence. Together the GFP, vimentin and α-SMA data indicate that fibroblasts are the primary cell type exhibiting activation of the procollagen α1(I)-GFP reporter gene. However, one caveat to this conclusion is whether there may be cells expressing GFP in the submucosa or muscularis that do express α-SMA and have myofibroblast phenotype, but that these cells are poorly detected by immunofluorescence with α-SMA antibodies because the levels of expression are lower than in smooth muscle cells within muscularis propria. To attempt to address this issue, we performed preliminary analyses in procollagen α1(I)-GFP/α-SMA-RFP dual reporter mice given one or two DSS cycles.

**Figure 9:**
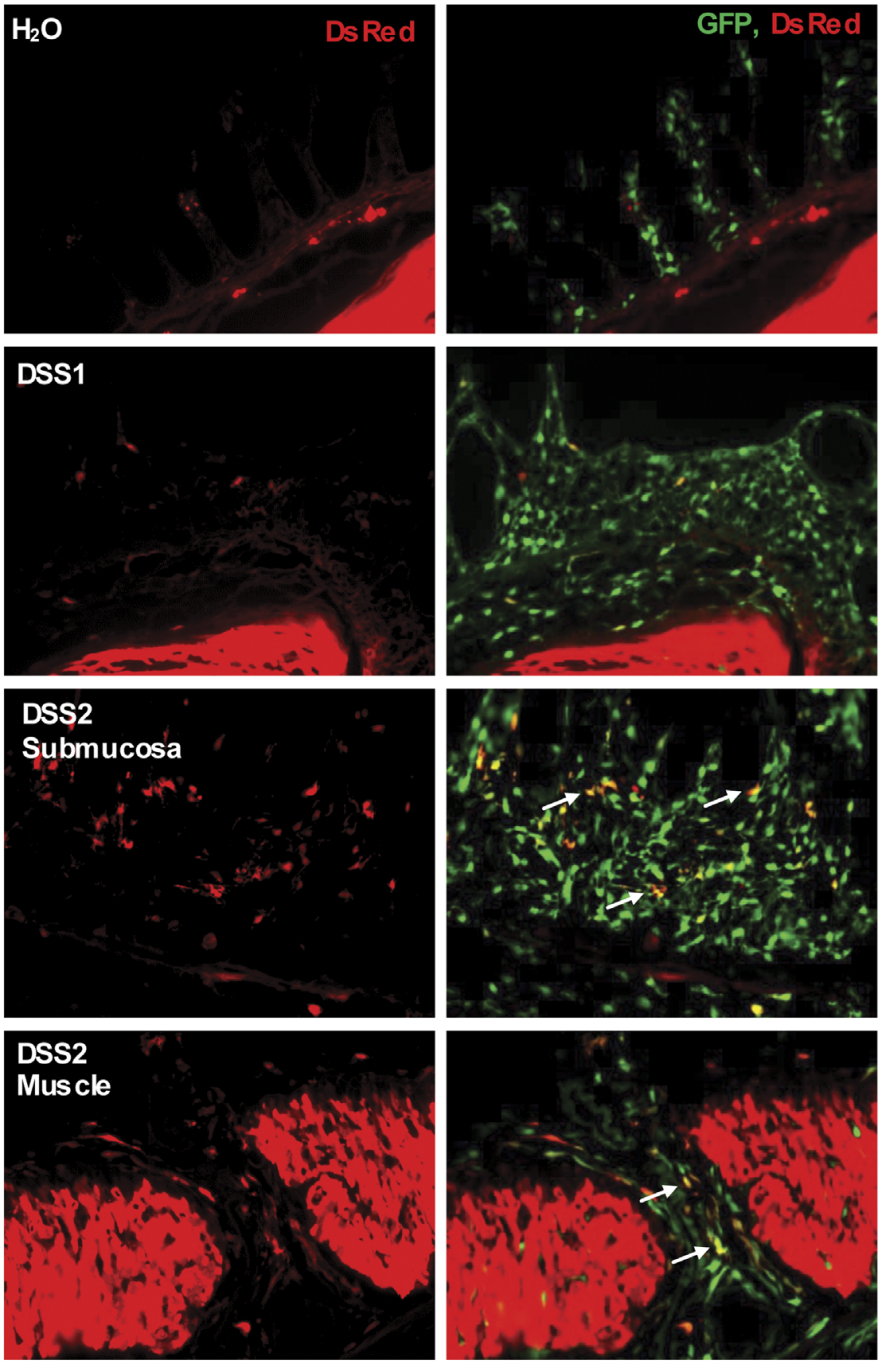
DSS induced procollagen-GFP^+^/α-SMA-RFP^+^ cells in mucosa, submucosa and muscularis layers in dual reporter mouse colon. Dual reporter gene transgenic mice were treated with one cycle of DSS (DSS1) or two cycles of DSS (DSS2), Fluorescence microscopy images indicate dramatic increased colocalization of GFP(+) cells with α-SMA-RFP(+) cells in mucosa and transmural fibrosis (white arrows).

As shown in Figure 9, in colon of water controls α-SMA-RFP reporter expression was abundant in muscularis propria and evident in muscularis mucosa, but little or no expression was detected in lamina propria and submucosa. One DSS cycle induced α-SMA-RFP reporter expression in mucosa and submucosa. In mice given two cycles of DSS, more cells expressing α-SMA-RFP reporter were detected in mucosa and submucosa. Evaluation of GFP on the same sections revealed overlap between many of the RFP positive cells and GFP although still many more GFP^+^ than RFP^+^ cells. Also, after two DSS cycles, regions of disorganized muscularis propria showed scattered α-SMA-RFP^+^ cells, which colocalized with procollagen α1(I)-GFP and a very small number of dual positive cells were detected in grossly normal muscularis propria. Together, these findings suggest that after DSS, there is a subpopulation of cells that co-express procollagen α1(I)-GFP reporter and α-SMA or α-SMA-RFP reporter.

### Procollagen α1(I) Reporter is Activated during TNBS Colitis

**Figure 10:**
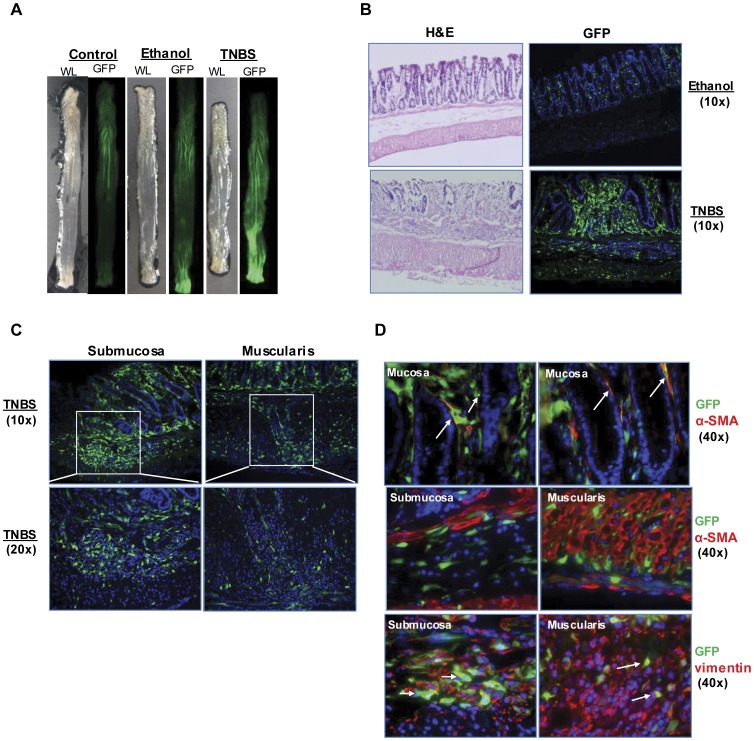
GFP reporter expression is induced in TNBS colitis model. (A) Representative white light and GFP images of whole colon from procollagen α1(I)-GFP reporter mice after one enema of TNBS versus ethanol or water control. Note increased GFP in distal colon of TNBS (and to a lesser extent, ethanol) treated mice. (B) Low power images of H&E and procollagen GFP activation obtained from control and TNBS treated mice colon (10X). (C) High power images demonstrate that GFP reporter expression is induced in submucosa and muscularis propria in colon of TNBS-treated mice. (D) High power images from immunostaining demonstrate colocalization of vimentin and GFP (white arrows) in submucosa and muscularis. Limited colocalization of α-SMA^+^ and GFP^+^ cells were detected in mucosa but not in submucosa and muscularis.

TNBS induced colitis is characterized by transmural inflammation of the colon that shares some histopathological features with human Crohn’s Disease [17]. Even early TNBS colitis is associated with T-cell-mediated inflammation [15], [18]. Visualization of entire colon revealed increased procollagen α1(I)-GFP reporter activation in the most distal portion of the colon in TNBS treated animals versus ethanol treated or untreated controls. This corresponds to the region exposed to enema. H&E staining of colon sections (Figure 10B) verified that one enema with TNBS given after skin sensitization caused transmural inflammatory infiltration and thickening of muscularis layer. Procollagen α1(I)-GFP expression was induced throughout lamina propria, submucosa and scattered cells within the muscularis propria. Similar to the DSS model, colocalization studies revealed that a majority of submucosal GFP^+^ cells are also vimentin^+^ and that GFP^+^/vimentin^+^ cells are evident in muscularis. A few cells in the mucosa expressed both GFP and α-SMA. In contrast, there was essentially no overlap between procollagen α1(I)-GFP reporter and α-SMA in submucosa or muscularis when evaluated by immunofluorescence.

## Discussion

A major goal of the current study was to assess wound healing and fibrogenic responses to acute and chronic DSS induced mucosal injury and inflammation based on changes in extent and site of ECM deposition. We compared responses to DSS in two inbred mouse strains commonly used to derive genetically manipulated mice and assessed the utility of a procollagen α1(I)-GFP reporter mouse as a readout for activation of a gene encoding a major ECM protein during DSS or TNBS induced disease. Our results provide important information relevant to use of the DSS model and a new reporter mouse to test impact of interventions or genetic manipulations on normal mucosal healing and fibrosis.

Our findings that males on both FVB-N and C57BL6 backgrounds exhibit more weight loss, more persistent hemoccult positivity and higher mortality/need for euthanasia demonstrate that males develop more serious disease or disease complications during the recovery period after DSS, especially after one cycle of DSS. Practically, such findings suggest that data in males and females need to be analyzed separately when evaluating impact of therapeutic/prophylactic interventions or genetic manipulations on disease severity, wound healing or fibrosis. However, lower amounts of DSS (2 or 2.5% of DSS) than the 3% DSS used here may improve survival in males and may be a useful strategy for investigators including males in their study. Our findings of sex-dependent differences in susceptibility to DSS are consistent with prior reports that male C3H/HeJ mice developed more severe DSS-induced colonic lesions [7] and that across a number of mouse strains, females were more resistant to DSS-induced disease [8]. Our findings are also relevant to emerging evidence that IBD susceptible loci including Toll-like receptor 8 reside on the X chromosome [19], [20].

In FVB-N mice, the most severe colitis and fibrosis was observed after 3 days of recovery following one cycle of DSS. By 7 days of recovery, DSS treated FVB-N mice showed reduced colitis and fibrosis scores which did not differ significantly from water controls. This suggests that FVB-N mice exhibit only transient increases in ECM deposition and mucosal healing between 3 and 7 days after one DSS cycle. This pattern of disease provides evidence that evaluation of the recovery period after one DSS cycle in FVB-N mice could be particularly useful to test whether particular interventions or genetic manipulations accelerate or improve mucosal healing, which is currently considered an important therapeutic and prognostic indicator in CD [21]. FVB-N mice subjected to one DSS cycle and recovery could also prove useful to explore differences in molecular or functional phenotypes of mesenchymal cells at times after DSS associated peak mucosal damage and ECM deposition versus times associated with mucosal healing and restoration of normal mucosal architecture. In FVB-N mice, two DSS cycles and a 7 day recovery period resulted in transmural increases in ECM in a subset of animals but overall did not consistently lead to more severe or sustained fibrosis. This suggests that FVB-N mice may need more DSS cycles to induce severe transmural fibrosis or may be resistant to development of fibrosis. Alternative mouse models of chronic intestinal inflammation and fibrosis exist including one or multiple cycles of intrarectal TNBS treatment [22]–[26] or salmonella infection [27]. Each of these models and the DSS model have strengths and limitations as models of IBD. However, the technical ease of the DSS model, and the transient fibrogenic changes and subsequent mucosal healing in FVB-N mice during recovery from one DSS cycle, provides a potentially useful and readily accessible model for initial testing of interventions or therapies that may impact these processes.

Our studies in procollagen α1(I)-GFP reporter mice aimed to test whether the reporter gene is activated during acute DSS-induced injury or more chronic inflammation and provide proof of principle as to whether levels or sites of reporter gene activation in whole colon or histologically could provide a useful readout of mesenchymal cell activation. Procollagen α1(I)-GFP mice have proved useful to investigate the cellular mechanisms of liver fibrosis [28], [29]. Increased expression and deposition of type I collagen is a major component of fibrosis in CD and intestinal stricture formation [25], [30]. Histology on procollagen α1(I)-GFP mice demonstrated that in contrast to FVB-N mice, mice on C57BL6 background did not show mucosal healing through 7 days of recovery after a single DSS cycle and disease progressively worsened to severe and transmural colitis after 2 DSS cycles. These findings are consistent with prior reports that C57BL6 mice do not show mucosal healing but develop chronic colitis after a single DSS cycle [11], [12]. Importantly, transmural colitis was associated with transmural activation of the procollagen α1(I)-GFP reporter gene, suggesting activation of mesenchymal cells to increase expression of a major component of fibrotic ECM. Strong and significant correlations between whole colon GFP intensity and histologic fibrosis scores, and between GFP mRNA and endogenous collagen α1(I) mRNA provide important evidence that procollagen α1(I)-GFP reporter activation provides a rapid and easy measure of activation of fibrogenic processes and induction of a major ECM gene, and could be potentially useful to study responses to interventions aimed at limiting fibrosis. We recognize that more studies will be required to definitively establish if procollagen α1(I)-GFP activation faithfully reflects development of fibrosis or increased expression or deposition of ECM proteins. We back-crossed the procollagen α1(I)-GFP mice onto the C57BL6 background to facilitate cross-breeding of this model with genetically manipulated mice on this same background in order to test genetic manipulations that may impact activation of this key fibrogenic gene. Confirmation that the procollagen α1(I)-GFP reporter is activated in the alternate TNBS and T cell driven model of colitis further supports the utility of the procollagen α1(I)-GFP reporter model. Histological findings that procollagen α1(I)-GFP expressing cells are located mainly in mucosa and submucosa after one DSS cycle and recovery, followed by transmural activation in cells throughout the bowel wall, including muscularis and serosa after two DSS cycles, demonstrate that the reporter will provide a useful tool to visualize and study the molecular or functional phenotypes of mucosal, submucosal and muscularis- or serosa-based mesenchymal cells that are activated to express this key fibrogenic gene.

Current views implicate myofibroblasts or modified smooth muscle cells as major mediators of transmural fibrosis in CD [1], [30]. However, it is important to note that one detailed study in tissues from patients with CD documented increases in vimentin^+^/SMA^−^ fibroblasts as well as vimentin^+^/SMA^+^ cells in regions of fibrosis in CD [4]. Our comparison of the cellular sites of procollagen α1(I)-GFP expression and α-SMA or vimentin immunoreactive cells indicates that in response to DSS or TNBS colitis, vimentin positive cells are by far the most abundant cell types exhibiting activation of procollagen α1(I)-GFP in mucosa, submucosa, serosa and, muscularis layers. This suggests that cells with fibroblast phenotype are the primary cell type activated to express procollagen α1(I) in the DSS and TNBS models. Similar conclusions were reached by Suzuki et al [12] who colocalized type I collagen with vimentin or α-SMA. After two DSS cycles, however, in both DSS and TNBS models, we did observe α-SMA^+^ cells with activated procollagen α1(I)-GFP reporter in the lamina propria but not submucosa or muscularis. This may suggest that mucosal rather than submucosal fibroblasts are activated to myofibroblast phenotype in both models because of injury initiated in epithelium and mucosa. Given that transmural activation of cells to myofibroblast phenotype is involved in fibrosis associated with IBD, this should be considered a limitation of the DSS and acute TNBS models in terms of relevance to CD fibrosis. However, it is also important to consider the possibility that immunofluorescence approaches may underestimate the number of myofibroblasts co-expressing reporter and α-SMA if levels of SMA are, for example, lower than in smooth muscle cells. Preliminary studies in a new dual reporter model expressing an α-SMA-RPF reporter as well as the procollagen α1(I)-GFP reporter suggest that this may be the case. In this model, dual labeled cells were observed in both submucosa and disorganized muscularis as well as mucosa. This emphasizes that characterization of mesenchymal cell types involved in injury or inflammation-induced fibrosis may require multiple approaches to definitively assess cell phenotype. Clearly more will need to be done to definitively identify the cell types expressing procollagen α1(I)-GFP reporter and their relevance to wound healing after acute injury or to transmural fibrosis associated with chronic inflammation. However, the procollagen α1(I)-GFP reporter provides a potentially useful and valuable system to apply flow cytometry based methods to compare, quantify and isolate cells showing activation of a key fibrogenic gene at different times during wound healing or fibrosis in DSS or other IBD and intestinal injury models. Fluorescence activated cell sorting of cells expressing the procollagen α1(I)-GFP reporter, combined with antibody based sorting for α-SMA or vimentin may provide a more complete or sensitive evaluation of the numbers of cells co-expressing GFP reporter and myofibroblast or fibroblast biomarkers at different stages of DSS or TNBS induced disease and fibrogenic changes. Such studies will also provide unique opportunities to define molecular and functional changes in different populations of fibrogenic cells during injury, fibrosis or therapeutic interventions. We should also emphasize that the visualization of GFP by fluorescence microscopy may complement and have benefits relative to localization of cell specific antigens on Sirius red or Masson’s Trichrome stained sections, because GFP is intracellular, while secretion and deposition of ECM in the extracellular compartment can make it difficult to definitively establish the precise cell types that are sources of ECM/collagen. The dual reporter model offers additional opportunities to compare, quantify and isolate α-SMA positive and α-SMA negative procollagen α1(I)-GFP expressing cells in DSS, TNBS or other intestinal injury and inflammation models. Our current preliminary studies provide an important first step towards more extensive characterization of this model and its value and validity for studies of the cellular and molecular basis of intestinal fibrosis.

In conclusion, the current study characterizes DSS-induced mucosal injury, and mucosal healing in FVB-N mice and DSS or TNBS-induced colitis and fibrosis in C57BL6 procollagen α1(I)-GFP reporter mice as an important step to use of these models to better understand mechanisms of mucosal healing and fibrosis. Our study demonstrates activation of the procollagen α1(I)-GFP reporter during acute mucosal injury or chronic, transmural inflammation. This provides an essential first step to further use of this model to better define cellular mediators and molecular phenotypes of mesenchymal cells, or potentially other cell types, that are activated during acute mucosal injury, or during chronic inflammation and progressive fibrosis, and to test interventions that may impact these processes.
