# Correction: Mucosal Healing and Fibrosis after Acute or Chronic Inflammation in Wild Type FVB-N Mice and C57BL6 Procollagen α1(I)-Promoter-GFP Reporter Mice

**DOI:** 10.1371/annotation/91f1d7f8-b09d-4067-943c-148e926b403b

**Published:** 2012-10-15

**Authors:** Shengli Ding, Kristen L. W. Walton, Randall Eric Blue, Kirk K. MacNaughton, Scott T. Magness, Pauline Kay Lund

The fourth author's name was spelled incorrectly. The correct name is: Kirk K. McNaughton.

The correct citation is: Ding S, Walton KLW, Blue RE, McNaughton KK, Magness ST, et al. (2012) Mucosal Healing and Fibrosis after Acute or Chronic Inflammation in Wild Type FVB-N Mice and C57BL6 Procollagen α1(I)-Promoter-GFP Reporter Mice. PLoS ONE 7(8): e42568. doi:10.1371/journal.pone.0042568 

